# Defining pharmacists' roles in disasters: A Delphi study

**DOI:** 10.1371/journal.pone.0227132

**Published:** 2019-12-26

**Authors:** Kaitlyn E. Watson, Judith A. Singleton, Vivienne Tippett, Lisa M. Nissen

**Affiliations:** 1 School of Clinical Sciences, Queensland University of Technology, Brisbane, Australia; 2 Institute of Health and Biomedical Innovation, Queensland University of Technology, Brisbane, Australia; Fukushima Medical University School of Medicine, JAPAN

## Abstract

**Introduction:**

Pharmacists are uniquely placed in the community to be of assistance to disaster-affected patients. However, the roles undertaken by pharmacists in disasters are identified based on their own experiences and networks. There is currently no definition or acknowledgment of pharmacists’ roles in disasters.

**Objective:**

To acquire consensus from an expert panel of key opinion leaders within the field of disaster health on pharmacists’ roles in disasters throughout the four disaster phases—prevention, preparedness, response, and recovery.

**Methods:**

A Delphi study consisting of three rounds of online surveys was utilised. Twenty-four key opinion leaders were contacted, with 15 completing all three rounds. The 15 expert panellists were presented with 46 roles identified in the literature and asked to rank their opinions on a 5-point Likert scale. This study used an international, all-hazard, and multijurisdictional approach. Consensus was benchmarked at 80% and any role which did not reach consensus was re-queried in the subsequent round. The third round provided the results of the Delphi study and sought commentary on the acceptance or rejection of the roles.

**Results:**

Of the 46 roles provided to the expert panel, 43 roles were accepted as roles pharmacists are capable of undertaking in a disaster. There were five roles for the prevention phase, nine for the preparedness phase, 21 for the response phase, and eight for the recovery phase. The experts were asked to prioritise the top five roles for each of the disaster phases. The three roles which did not make consensus were deemed to be specialised roles for disaster pharmacists and not generalisable to the broader pharmacy profession.

**Conclusion:**

This study identifies pharmacists’ roles in disasters which have been accepted by the international disaster health community. The international key opinion leaders recommended that pharmacists could be undertaking 43 roles in a disaster, however, this is dependent on individual jurisdiction considerations. Pharmacy professional associations need to advocate to policymakers for legislative support and to ensure pharmacists are equipped with the training and education required to undertake these roles within specific jurisdictions.

## Introduction

Pharmacists are uniquely positioned during disasters to provide healthcare continuity and medication management to affected communities. It has been acknowledged that pharmacists are the most widely distributed healthcare professional, being more accessible than supermarkets, banks, or medical centres.[1] However, pharmacists’ roles in disasters both formal and informal are currently undefined or acknowledged.

Anecdotally, pharmacists have been undertaking various tasks in disasters in an ad-hoc fashion to assist their communities.[2–5] However, pharmacists’ roles in disasters did not become of significant interest in the literature until the events of September 11, 2001 in the United States (US).[6] Prior to 2001, accepted roles for pharmacists generally focused on their contributions to logistics and supply chain management.[7] Post 2001, pharmacists began to be recognised for their informal contributions in patient care and public health during disasters, although their primary purpose and formal role was still logistics.[6, 8]

The strength that pharmacists provide during disasters, is their positioning in the community. They are considered community landmarks or communication hubs for patients and are typically the first healthcare service to resume operations after a disaster has occurred.[9, 10] Roles undertaken by pharmacists in disasters are within their scope of practice and are typically an extension of their daily duties (i.e. continuity of medication management). For example, patients tend to evacuate their homes without taking their medications, prescriptions, money or identification.[8] In this circumstance during a disaster, with the collapse of community healthcare services, pharmacists can provide a limited emergency supply of the patient’s chronic disease medications. [9–11] This can alleviate the healthcare burden on overcrowded emergency rooms and free up doctors and nurses to treat higher-acuity patients.

Hurricane Katrina, a Category Four hurricane, significantly affected parts of the US in 2005[12] and highlighted the impact pharmacists can have in the response and recovery of a community following a disaster. It was found pharmacists performed many duties in the wake of Hurricane Katrina and fulfilled additional roles in the absence of other healthcare professionals.[13] Some of the duties pharmacists performed following Hurricane Katrina included:[13–17]

- triaging services within evacuation centres (separating those patients needing to see a doctor from those who simply needed a prescription refill, and identifying and referring individuals to allied health professionals),
- taking medication histories,
- providing vaccinations,
- performing basic medical checks,
- mixing intravenous medications,
- providing consultations on wound infections,
- assisting with major traumas,
- assessing the contamination risk of medications brought in, and,
- pill identification.

Most these roles identified during Hurricane Katrina are services pharmacists provide daily but become significantly more important during disasters when there is a potential collapse of community healthcare services, or affected communities become cut off from outside assistance. In recognition of the crucial role pharmacists could play in alleviating the burden of low-acuity non-communicable disease patients, Alabama State (US) temporarily extended (during Hurricane Katrina) its ‘emergency supply’ rule. This allowed pharmacists to prescribe a 30-day emergency supply (increased from the everyday 3-day emergency supply rule) of chronic disease medications for disaster-affected patients without a prescription. Alabama State (US) also permitted the use of out-of-state volunteer pharmacists to assist in the relief efforts.[18, 19]

In November 2016, the Thunderstorm Asthma event affected the city of Melbourne, Australia. The Inspector-General for Emergency Management (IGEM) review coordinated by the Victorian State Government of the thunderstorm asthma emergency response, found community pharmacies and pharmacists were essential in the response and should be better integrated into disaster management teams for effective coordinated responses.[20] Pharmacists were integral in providing medication management for this event, due to the volume and accessibility of pharmacies in the communities, patients were able to get assistance quickly. This is highlighted in Finding Six of the IGEM report:

*“IGEM finds that on 21–22 November 2016 community pharmacies played a central role in meeting community needs during the thunderstorm asthma event. Given their community focus and their geographic coverage, community pharmacies can provide valuable support to the management of health emergencies or emergencies with health impacts.”[20]* ^*(p*.*36)*^

The literature has highlighted the valuable assistance pharmacists can provide in ensuring continuity of medication care. However, the formal acknowledgment or translation of these pharmacists’ roles into disaster health management has yet to be achieved beyond the established pharmacists’ role in logistics. To date, pharmacists have been operating their pharmacy disaster management response with limited formal guidelines to follow, separately to the coordinated health response. The purpose of this study was to convene an expert panel of key opinion leaders to discuss and begin to define and acknowledge pharmacists’ roles in disasters. Specifically, the aim was to obtain consensus on pharmacists’ roles within each of the disaster phases–prevention, preparedness, response, and recovery (PPRR). This study begins the process of formally acknowledging and providing acceptance from the international disaster health community on the roles undertaken by pharmacists in disasters. The potential pharmacists’ roles were identified from the literature[13–17] and a larger study conducted by the research team on pharmacists’ roles in disasters which included surveys and interviews with international disaster health professionals.[9]

## Methods

### Data collection and participant recruitment

A Delphi study is a process of gathering information through a series of survey rounds and is commonly used to obtain consensus on matters.[21] There are currently no universally accepted parameters for completing a Delphi study. However, it is generally agreed that, rounds should continue until an adequate consensus level is reached or the survey results reach stability with panellists no longer revising their rankings–typically achieved after three rounds.[21] The Delphi procedure should involve ‘controlled feedback’ providing the panellists a summary of the comments made and a simple statistical summary of the entire panel’s position.[21, 22] The purpose of feedback and including both the statistical measure and qualitative comments, allows for the individual panellists to determine where their response lies relative to the overall group’s and can assist in their revision of their ranking in future Delphi rounds.[21] The Delphi technique used should be systematic and transparent in its findings of reaching consensus.

This Delphi study asked the participants to take an international, all-hazard, and multijurisdictional approach to disasters and evaluate pharmacists at the level of the profession rather than referring to a specific employment context. Since, the study was international in scope, panellists were asked not to restrict the pharmacist’s roles based on their current jurisdiction’s legalisation framework. The researchers sought to investigate, whether the panellists perceived that pharmacists have the expertise, skills, and knowledge to undertake the potential roles in all-types of disasters.

Twenty-four national and international opinion leaders were identified by the research team as experts on health aspects of disaster management, advancing practice of pharmacy, and knowledge of pharmacists’ roles in disasters. These experts were identified from various international and Australian non-governmental organisations, government, pharmacy, military, public health, and disaster management agencies and were recruited through snowball and purposive sampling. The utilisation of snowball sampling techniques was performed to recruit additional potential panel members and account for the potential inherent bias of investigator selection. The experts were identified as leaders of their respective agencies and influential in matters of disaster management and advancing pharmacy practice. The countries included in this study were New Zealand, United Kingdom, Pan America, Europe, and Australia. The experts were recruited for their expertise on the subject matter and not specifically for their country of origin as many of the panellists have international interests. Initially, 24 opinion leaders were contacted twice to invite them to participate on the panel. Potential participants were provided with the participant information sheet. Of the 24 opinion leaders contacted, 15 consented and completed the three rounds of surveys. Consent was implied with submission of the completed surveys. Ethics was obtained from the Queensland University of Technology human research ethics committee, approval number 1700000106.

The Delphi panel were presented with 45 initial roles in Round 1 of this study. These 45 roles were identified from the literature as roles pharmacists were already undertaking in various jurisdictions[13–17] and roles identified from a larger study conducted by the research team which included surveys and interviews with international disaster health professionals.[9] The panellists were invited to suggest any additional roles they deemed appropriate which were presented to the expert panel in the subsequent rounds. There was one additional role added after Round One by a panellist, bringing the total number of roles assessed by the experts to 46 roles. Each of the 46 potential roles were categorised according to where in the PPRR cycle they would fit operationally. The consensus benchmark was set at 80%, as has been used in previous Delphi Studies on health outcomes and disaster management.[23, 24] For Round One, roles which received an ‘agree or strongly agree’ rating by at least 80% of the expert panel were accepted as having reached consensus and indicated agreement that pharmacists were perceived to be capable of undertaking these roles in a disaster. Roles which fell below 69% were required to confirm removal using a dichotomous ‘yes’ or ‘no’ question in the second round. Panellists were asked to provide a reason why pharmacists could not undertake this role. Roles which fell in between this range of 70%-79% were presented back to the panel in the second round with a four-point Likert scale, asking for panellists to agree or disagree with the role. Based on feedback from the panellists in round one, one new role was added in for subsequent rounds increasing the 45 potential roles to 46. The results from Round One were presented back to the panellists as ‘controlled feedback’[21, 22] and the experts were asked to revise their rankings of the 46 roles and to provide commentary. The second round preceded in the same manner as the first with all 15 participants providing their revised rankings and comments. For the final round, as the results had not changed between the first two rounds, the participants were provided with the final results and asked to confirm/accept them and provide any final comments.

### Data analysis

The survey Likert scale responses were exported from KeySurvey^®^ into IBM^®^ SPSS^®^ Statistical software version 25. Frequencies of each role in the Likert scale responses were analysed. The five-point Likert scales were trichotomized into a three-point Likert scale, with ‘disagree’ representing ‘1–2’, ‘neutral’ remaining ‘3’ and ‘agree’ representing ‘4–5’. Consensus was reached if 80% of the participants scored ‘4–5’ representing ‘agree’. This was similar in the second round which utilised a four-point Likert scale, however, the option of a neutral ‘3’ response was removed.

The qualitative comments provided by the experts were presented back to the panellists as arguments for and against pharmacists undertaking that specific role in disasters in the proceeding rounds and in the final results. This allowed the panellists to revise their rankings based on the ‘controlled feedback’ provided. It is suggested the survey feedback process should continue until either consensus is reached or the panellists stop revising their answers.[22] Stability was reached for this Delphi Study within the three rounds, as the experts no longer continued to revise their rankings and 43 roles reached consensus.

## Results

The expert panel consisted of key opinion leaders from international organisations considered experts in disaster health management and/or pharmacy. The original makeup of the potential panellist group contacted was evenly distributed: 33% pharmacy background, 33% disaster management, government, and medical background, and 33% experts with extensive experience in both backgrounds. Table 1 outlines the composition of the expert panel and self-identified background of the 15 panellists.

**Table 1:** Delphi study expert panel composition.

*Self-identified Background*	n (%) *
*Disaster Management, Government and Medical*	6 (40%)
*Pharmacy*	5 (33%)
*Both*	4 (27%)

*Total N = 15

The panellists were then asked to identify the different perspectives in which they were approaching the Delphi study. They were able to tick all perspectives that applied to them, thus increasing the denominator from the 15 panellists to 20 differing perspectives. The breakdown for the panellists’ perspectives were 15% (3/20) from disaster and emergency services, 30% (6/20) from government and policy perspective, 10% (2/20) were ‘other’ (disaster management and consultancy), and 45% (9/20) were from pharmacy. Of 14 experts who provided their age, the median age of the expert panel was 50.5 years (IQR 16.25 years), with the oldest participant being 72 years and the youngest 36 years old. There was seven females and eight males.

Out of 46 roles presented to the expert panel, consensus was reached on 43 roles. The final 43 roles deemed appropriate for pharmacists to undertake in a disaster are listed below, categorised according to each disaster PPRR phase. The five roles in the prevention phase are listed in Table 2, the nine roles in the preparedness phase are listed in Table 3, the 21 roles in the response phase are provided in Table 4, and the eight roles in the recovery phase are listed in Table 5. Each Delphi round results are presented in the Supporting Information (S1–S4 Tables).

**Table 2:** Final Consensus on five roles pharmacists are capable of undertaking in the prevention phase of a disaster.

**Prevention/Mitigation—reduce the health risks posed by hazards**
**Administer vaccinations**
**Educate the public on reducing the spread of communicable diseases/infections**
**Tailored 'point of care' messaging to chronic disease patients**
**Ensuring patients are aware of their increased risk of adverse health outcomes in a disaster**
**Optimising medication supplies for chronic disease management**

**Table 3:** Final Consensus on nine roles pharmacists are capable of undertaking in the preparedness phase of a disaster.

**Preparedness—ensure timely and effective response systems are in place**
**Ensuring uninterrupted supply of medications in a disaster**
**Knowing how to access national pharmaceutical stockpiles and who to contact if necessary**
**Develop business continuity plans that include disaster management to ensure sustainability of service**
**Developing drug algorithms and treatment guidelines to determine drug choice based on co-morbidities in the event of bio terrorism (e.g. Anthrax, Plague, Tularaemia—requiring antibiotics/prophylaxis measures)**
**Being a part of local/state/national disaster preparedness health meetings—providing medication management advice**
**Being a part of the local community disaster management teams to involve pharmacy in coordinated response**
**Maintain systems and process for the reconciliation and security of controlled drugs (e.g. morphine, oxycodone, etc.)**
**Have systems in place to secure cold chain lines**
**Develop a list of at-risk patients in their community**

**Table 4:** Final Consensus on 21 roles pharmacists are capable of undertaking in the response phase of a disaster.

**Response—action in disaster/emergency**
**Coordinating logistics of medications and medical supplies for patients with chronic diseases**
**Rationing limited supplies of medications**
**Assisting with the release and allocation of national stockpiles if required in pandemic or emergency**
**Triage of low-acuity patients. (e.g. medication reconciliation, patient medical history, referring to physician for further assessment or to pharmacist for refill of lost medications)**
**Institute cardiopulmonary resuscitation (CPR)**
**Provide wound care and first aid for minor ailments**
**Providing one off medication emergency supply refills for up to 30 days during the declared disaster**
**Continue provision of chronic disease medications**
**Dispense medications and other necessary medication-related items to affected members of the community (prescription, over-the-counter medications, inhalers, etc.)**
**Dispense general health pharmacy items to affected members of the community (toiletries, nappies, bandages, incontinence pads, water, etc.)**
**Making therapeutic substitutions for drugs available on limited formularies without prior authorisation**
**Counselling patients on how to use and take medications**
**Prescribing and administering vaccinations (e.g. tetanus, antidote/prophylaxis to bio-terrorism agent following state public health disaster protocols)**
**Attend clinical ward rounds to provide pharmacist expertise on medical patients**
**Prescribe medication needs of low-acuity patients in hospital**
**Medication identification and safety assessment**
**Monitoring the chronic disease(s) of at-risk individuals to minimise exacerbation**
**Advocate pharmacy’s role during an event**
**Maintain media liaison on medication issues**
**Decide on the appropriateness of donated medications and other supplies**
**Pharmacists should engage the pharmacy student workforce to backfill duties (dispensing, inventory, etc.), freeing up pharmacists to perform more clinical roles in a disaster.**

**Table 5:** Final Consensus on eight roles pharmacists are capable of undertaking in the recovery phase of a disaster.

**Recovery—returning to 'normal' business and beyond**
**Check on the health needs of the local community**
**Re-establish normal stock levels, destroy contaminated stock appropriately**
**Restock emergency/ disaster kits for next disaster event**
**Identify and prioritise vulnerable patients in local community**
**Restore order to patient records and drug records, if manually written due to power outages**
**Document what worked and what didn't in the disaster response and change disaster plans accordingly**
**Participate in post-disaster research/reports**
**Inform local disaster management reports on pharmacy response improvements**

Consensus was not reached on the following three roles and therefore they were removed from the overall list of accepted roles pharmacists can formally undertake in disasters. The roles removed were:

1. Develop educational tools for health professionals on preparedness, signs and symptoms and drug treatments for CBRN (chemical, biological, radiological and nuclear) weapons
2. Making dose adjustments to existing therapeutic regimens where clinically necessary
3. Pharmacists providing behavioural and mental health support to their patients, customers, and staff following a disaster

The expert panel were asked to prioritise the top five pharmacists’ roles for each of the PPRR phases. Their prioritisation of pharmacists’ roles is provided in Table 6.

**Table 6:** Role prioritisation of pharmacists’ roles for the disaster PPRR phases obtained in round 1 of the Delphi study by the expert panel.

Priority	Prevention	Preparedness	Response	Recovery
**1^st^**	Optimising medication supplies for chronic disease management	Ensuring uninterrupted supply of essential medications in a disaster	Dispense medications and other necessary medication-related items to affected members of the community (prescription, over-the-counter medications, inhalers)	Re-establish normal stock levels, destroy contaminated stock appropriately
**2^nd^**	Administer vaccinations	Have systems in place to secure cold chain lines	Counselling patients on how to use and take medications*	Restock emergency/ disaster kits for next disaster event
**3^rd^**	Educate the public on reducing the spread of communicable diseases/infections	Being a part of the local community disaster management teams to involve pharmacy in coordinated response	Coordinating logistics of medications and medical supplies for patients with chronic diseases*	Check on the health needs of the local community**
**4^th^**	Ensuring patients are aware of their increased risk of adverse health outcomes in a disaster	Knowing how to access national stockpiles if necessary	Providing one off medication emergency supply refills for up to 30 days during the declared disaster	Identify and prioritise vulnerable patients in local community**
**5^th^**	Tailored 'point of care' messaging to chronic disease patients	Being a part of local/state/national disaster preparedness health meetings—providing medication management advice	Assisting with the release and allocation of national stockpiles if required in pandemic or emergency	Restore order to patient records and drug records, if manually written due to power outages

*Tied for second prioritisation

**Tied for third prioritisation

## Discussion

This Delphi study followed the guidelines outlined in a systematic review by Sinha, Smyth and Williamson.[25] This method provided a systematic and transparent approach resulting in rigorous findings. Panellists recommended 43 out of 46 possible roles that they believe pharmacists are capable of undertaking across the PPRR cycle. Utilising a Delphi study technique to evaluate pharmacists’ capabilities is not a new endeavour. In 2012, Kennie-Kaulbach et al. utilised a modified-Delphi study to appraise pharmacists’ competencies in their provision of primary health care.[26] Delphi studies have also been widely used across the disaster management research space.[24, 27, 28]

This study utilised an international, all-hazard, multijurisdictional approach. Disaster literature has identified that disasters are transcending boundaries (i.e. geographical, organisational, academic), making them complex in their impact on the communities, the organisations involved, and in the response required.[29–31] Healthcare systems, infrastructure, and resources including pharmacy need to be prepared for any type of emergency situation. This Delphi study discussed pharmacy at the international profession level as often disasters are multijurisdictional and require the assistance of pharmacists from different employment contexts and jurisdictions. Thus, this study sought to begin to outline pharmacists’ scope of practice in disasters and disaster health management.

Despite reaching some consensus on the scope of potential roles for pharmacists, not all countries have supportive legislative frameworks and polices to facilitate all 43 roles when the next disaster impacts their community. A review of legislation as well as support from pharmacy organisations and disaster management organisations is required to collaboratively instigate these roles across the PPRR cycle. The acknowledged pharmacists’ roles are designed to be undertaken in a cooperative team dynamic in partnership with other stakeholders. This could be facilitated by better integration and utilisation of pharmacists into disaster teams.

This study provides evidence to policymakers on the acknowledgment by international key opinion leaders in the disaster health community of the accepted roles pharmacists are capable of undertaking in disasters. However, it is up to the pharmacy profession to instigate the capability of these pharmacists’ roles through training for specialist roles and expanding pharmacy operational environments. It is up to the pharmacy professional bodies to take a disaster pharmacy proposal to the government about how more extensive involvement of pharmacists in communities and on deployment could be achieved. Together, pharmacy professional bodies and policymakers can ensure the gaps identified in pharmacy legislation and knowledge are met, fully utilising pharmacists’ scope of practice and better integrating primary healthcare providers within the disaster management structure.

There were three posited roles for pharmacists which generated considerable debate in this study. The potential role for pharmacists in CBRN education was highlighted by the panellists as perhaps a specialist role for pharmacists requiring additional training and therefore was not adopted in the definition and consensus. This is corroborated by pharmacists working within the Office of Public Health Preparedness teams in developing medication management guidance for CBRN disasters for the US in the last 10 years.[32] However, in Canada, community pharmacists are identified as one of the first professionals to be potentially faced with a CBRN incident and therefore they are included in the CBRN first responder basic training program.[33] The training outlines that being a CBRN first responder is not intervening in the incident but recognising the signs and symptoms and responding appropriately.[33] For pharmacists specifically, the signs and symptoms of biological attacks may present in pharmacies as an unusual acceleration of people coming in with similar ‘flu-like’ symptoms. Terriff and Tee reported in 2001 that pharmacists have a role in the rapid dispersal of antidotes and information on both treatment and prophylaxis measures in a bioterrorism event.[34] They suggest many of the medications used in the treatment and prophylaxis of bioterrorism agents are stocked in pharmacies.[34] It has been suggested pharmacists do have a role in the planning for these events by keeping the information on the medications and vaccines used in a CBRN disaster up-to-date for other health professionals.[34, 35]

The potential pharmacists’ role in providing dose adjustment in disasters did not reach consensus. The panellists identified this role requires close collaboration with the prescriber which they identified not all current pharmacy models in a disaster are capable of achieving. However, some countries have integrated dose-adjustment as part of the everyday responsibilities of pharmacists.[36]

Pharmacists’ potential role in providing mental health and behaviour first-aid support did not reach consensus and therefore was not adopted in the definition. The discussion of this role by the panellists was very split. Some of the panellists believed mental health support following a disaster should be left up to the professionals. Whereas, the other half of the panellists believed this role was an extension of the mental health support role pharmacists fulfil in the community daily. In disasters, the principle of psychological first-aid or behavioural support is similar to the daily mental health support role in the recognition of warning signs and referral to appropriate healthcare services but is specific to the type of trauma experienced.[37] Both sides of the panellist’s debate are accurate and reflect the need for referral onto appropriate healthcare services. Research has suggested pharmacists are ideally placed as potential first responders for the screening of those requiring professional assistance for mental health crises, as they assist with mental health conditions as part of their daily practice.[38] In Australia, the Mental Health First Aid^®^ training program has begun to be incorporated into undergraduate pharmacy degree curricula. The aim of this program is to educate and upskill pharmacists in the recognition of a mental health crisis and to identify the appropriate health services and professionals to which to refer their patients.[39]

The expert panel revised their ranking and came to consensus on the pharmacists’ role in CPR. Pharmacists as members on CPR teams are often not the professional doing the compressions, with the skill sets of other health professionals better equipped to perform that task. Pharmacists can provide important drug information, perform dose calculations, prepare drugs for administration, and record the drugs used during a code blue.[40] However, there may be instances where pharmacists are the most qualified healthcare professional available to instigate CPR and thus should be competent to perform it until assistance arrives. In Australia, to become a registered pharmacist it is a requirement to have a current certification in CPR and first aid. This certificate then must be maintained for any pharmacist qualified to administer vaccinations.[41–43] The CPR and vaccination roles in Australia are not disaster-specific, referring to everyday situations.

The expert panellists defined the roles pharmacists should be undertaking within the four disaster phases–PPRR. They acknowledged the principle purpose of pharmacists in disasters is to ensure continuity of care which encompasses many different facets, from the logistics of obtaining medications to providing direct patient care and expert advice to other healthcare professionals. Not every jurisdiction can implement all 43 roles immediately; it requires a collaborative effort from all stakeholders. To undertake these roles effectively pharmacists should work within the coordinated disaster management and public welfare team structure.

### Limitations

A limitation of this Delphi study was the time commitment of key opinion leaders. Many of these key opinion leaders lead very busy lives and jobs and therefore some of them could not commit to partaking in a three-round Delphi panel. However, of the key opinion leaders approached, 62.5% (15/24) completed all three rounds. Another limitation of this study is that the opinion leaders were identified and selected by the investigators with the majority being Australian and so there may have been potential inherent bias. To reduce this possibility of inherent bias the investigators contacted national and international experts and utilised snowball sampling techniques for the identification of additional panel members by the experts. This research has highlighted the acknowledgment of pharmacists’ roles and has begun to identify their scope of practice in disasters. However, due to the multijurisdictional study design utilised, the limitations within individual jurisdictions was not explored. Further research identifying the different legislative considerations between jurisdictions and exploring potential need for additional education or trainings within individual jurisdictions could be investigated.

## Conclusion

The international key opinion leaders have acknowledged pharmacists’ roles in disasters throughout the disaster PPRR cycle. They have formally accepted 43 roles which extend beyond the well-established logistics role. Pharmacy professional bodies need to provide trainings for specialised disaster pharmacists’ roles and advocate for supportive disaster pharmacy legislation. Together, pharmacy professional bodies and policymakers can provide better integration of pharmacist roles in disaster management teams, whether assisting in the community or on deployment.

## Supporting information

S1 Table Round 1 Delphi study survey results utilising a five-point Likert scale, roles were divided into the PPRR disaster phases taking an all-hazard approach. (DOCX)

S2 Table Round 2 Delphi survey utilising a four-point Likert scale and the panellists’ comments, roles were divided into the PPRR disaster phases taking an all-hazard approach and consensus is indicated against each role. (DOCX)

S3 Table Final round Delphi study qualitative comments on roles which had reached consensus for pharmacists’ roles across the disaster PPRR phases utilising an all-hazard approach. (DOCX)

S4 Table Final round Delphi study survey on roles which had not yet reached consensus in the previous two rounds and included the comments from the panellists. (DOCX)

## Abbreviations

CBRN: chemical, biological, radiological and nuclear
CPR: cardiopulmonary resuscitation
IGEM: Inspector-General for Emergency Management
PPRR: prevention, preparedness, response, and recovery
US: United States
