# Supplementary material for: Defining pharmacists' roles in disasters: A Delphi study
Source: PLoS One. 2019 Dec 26;14(12):e0227132. doi: 10.1371/journal.pone.0227132 (PMC6932796; doi:10.1371/journal.pone.0227132)
Supplement: S1 Table — (DOCX) [file pone.0227132.s001.docx]

S1 Table: Round 1 Delphi study survey results utilising a five-point Likert scale, roles were divided into the PPRR disaster phases taking an all-hazard approach

| Roles | Strongly Disagree & Disagree | Neutral | Strongly Agree & Agree |
| --- | --- | --- | --- |
| Prevention/Mitigation - reduce the health risks posed by hazards (n=15) | | | |
| Administer vaccinations | 0 | 1 | 14 |
| Educate the public on reducing the spread of communicable diseases/infections | 0 | 0 | 15 |
| Tailored 'point of care' messaging to chronic disease patients | 0 | 0 | 15 |
| Ensuring patients are aware of their increased risk of adverse health outcomes in a disaster | 0 | 1 | 14 |
| Optimising medication supplies for chronic disease management | 0 | 1 | 14 |
| Preparedness - ensure timely and effective response systems are in place | | | |
| Ensuring uninterrupted supply of medications in a disaster | 0 | 1 | 14 |
| Knowing how to access national stockpiles if necessary | 1 | 2 | 12 |
| Develop business continuity plans that include disaster management to ensure sustainability of service | 0 | 1 | 14 |
| Developing drug algorithms and treatment guidelines to determine drug choice based on co-morbidities in the event of bio terrorism (e.g. Anthrax, Plague, Tularaemia - requiring antibiotics/prophylaxis measures) | 3 | 2 | 10 |
| Being a part of local/state/national disaster preparedness health meetings - providing medication management advice | 0 | 1 | 14 |
| Being a part of the local community disaster management teams to involve pharmacy in coordinated response | 0 | 1 | 14 |
| Develop educational tools for health professionals on preparedness, signs and symptoms and drug treatments for CBRN (chemical, biological, radiological and nuclear) weapons | 3 | 2 | 10 |
| Maintain systems and process for the reconciliation and security of controlled drugs (e.g. morphine, oxycodone) | 1 | 0 | 14 |
| Have systems in place to secure cold chain lines | 0 | 3 | 12 |
| Develop a list of at-risk patients in their community | 1 | 1 | 13 |
| Response - action in disaster/emergency | | | |
| Coordinating logistics of medications and medical supplies for patients with chronic diseases | 1 | 1 | 13 |
| Rationing limited supplies of medications | 0 | 3 | 12 |
| Assisting with the release and allocation of national stockpiles if required in pandemic or emergency | 1 | 1 | 13 |
| Triage of low-acuity patients. (e.g. medication reconciliation, patient medical history, referring to physician for further assessment or to pharmacist for refill of lost medications) | 1 | 1 | 13 |
| Institute cardiopulmonary resuscitation (CPR) | 3 | 3 | 9 |
| Provide wound care and first aid for minor ailments | 2 | 2 | 11 |
| Providing one off medication emergency supply refills for up to 30 days during the declared disaster | 1 | 1 | 13 |
| Continue provision of chronic disease medications | 1 | 0 | 14 |
| Dispense medications and other necessary medication-related items to affected members of the community (prescription, over-the-counter medications, inhalers) | 0 | 0 | 15 |
| Dispense general health pharmacy items to affected members of the community (toiletries, nappies, bandages, incontinence pads, water) | 0 | 0 | 15 |
| Making therapeutic substitutions for drugs available on limited formularies without prior authorisation | 2 | 1 | 12 |
| Making dose adjustments to existing therapeutic regimens where clinically necessary | 3 | 1 | 11 |
| Counselling patients on how to use and take medications | 0 | 0 | 15 |
| Prescribing and administering vaccinations (e.g. tetanus, antidote/prophylaxis to bio-terrorism agent following state public health disaster protocols) | 2 | 1 | 12 |
| Attend clinical ward rounds to provide pharmacist expertise on medical patients | 0 | 1 | 14 |
| Prescribe medication needs of low-acuity patients in hospital | 2 | 1 | 12 |
| Medication identification and safety assessment | 0 | 0 | 15 |
| Monitoring the chronic disease(s) of at-risk individuals to minimise exacerbation | 1 | 4 | 10 |
| Advocate pharmacy’s role during an event | 0 | 1 | 14 |
| Maintain media liaison on medication issues | 1 | 1 | 13 |
| Decide on the appropriateness of donated medications and other supplies | 1 | 1 | 13 |
| Recovery - returning to 'normal' business and beyond | | | |
| Provide Mental Health support | 3 | 3 | 9 |
| Check on the health needs of the local community | 0 | 3 | 12 |
| Re-establish normal stock levels, destroy contaminated stock appropriately | 0 | 1 | 14 |
| Restock emergency/ disaster kits for next disaster event | 0 | 1 | 14 |
| Identify and prioritise vulnerable patients in local community | 0 | 2 | 13 |
| Restore order to patient records and drug records, if manually written due to power outages | 1 | 1 | 13 |
| Document what worked and what did not in the disaster response and change disaster plans accordingly | 0 | 0 | 15 |
| Participate in post-disaster research/reports | 0 | 0 | 15 |
| Inform local disaster management reports on pharmacy response improvements | 0 | 0 | 15 |
