# Supplementary material for: Defining pharmacists' roles in disasters: A Delphi study
Source: PLoS One. 2019 Dec 26;14(12):e0227132. doi: 10.1371/journal.pone.0227132 (PMC6932796; doi:10.1371/journal.pone.0227132)
Supplement: S2 Table — (DOCX) [file pone.0227132.s002.docx]

S2 Table: Round 2 Delphi survey utilising a four-point Likert scale and the panellists’ comments, roles were divided into the PPRR disaster phases taking an all-hazard approach and consensus is indicated against each role

| Roles | Consensus  Reached | Comments |
| --- | --- | --- |
| Prevention/Mitigation - reduce the health risks posed by hazards (n=15) | | |
| Administer vaccinations | ✓ | "Not sure how to 'read' the word administering here. In terms of managing ok but in terms of dispensing/applying I would say no"  "This is a clearly a critically role and is shared with other members of the healthcare team."  "Yes, this is a role that pharmacists are already fulfilling in non-disaster times. It is important though to ensure adequate training and clear procedures for commissioning."  "This depends on the pharmacist’s other duties and the level of support they receive. When they are the only pharmacist, they may be too busy doing other roles which can't be done by other professions." |
| Educate the public on reducing the spread of communicable diseases/infections | ✓ | "Depending on the time on deployment and the other competing demands"  "Pharmacists often spend much face-to-face time with their patients. This is a perfect opportunity to provide this type of information" |
| Tailored 'point of care' messaging to chronic disease patients | ✓ | "Pharmacy professionals are well-placed to undertake such role."  "As a part of medication counselling"  "Pharmacists often spend much face-to-face time with their patients. This is a perfect opportunity to provide this type of information." |
| Ensuring patients are aware of their increased risk of adverse health outcomes in a disaster | ✓ | "Pharmacists often spend much face-to-face time with their patients. This is a perfect opportunity to provide this type of information."  "As a part of medication counselling"  "Important role to increase resilience of the health system in disasters and emergencies" |
| Optimising medication supplies for chronic disease management | ✓ | "no question about this one." |
| Preparedness - ensure timely and effective response systems are in place | | |
| Ensuring uninterrupted supply of medications in a disaster | ✓ | "To attempt to ensure it, anyway"  "where possible" |
| Knowing how to access national stockpiles if necessary | ✓ | No comments made |
| Develop business continuity plans that include disaster management to ensure sustainability of service | ✓ | No comments made |
| Developing drug algorithms and treatment guidelines to determine drug choice based on co-morbidities in the event of bio terrorism (e.g. Anthrax, Plague, Tularaemia - requiring antibiotics/prophylaxis measures) | ✓ | No comments made |
| Being a part of local/state/national disaster preparedness health meetings - providing medication management advice | ✓ | No comments made |
| Being a part of the local community disaster management teams to involve pharmacy in coordinated response | ✓ | No comments made |
| Develop educational tools for health professionals on preparedness, signs and symptoms and drug treatments for CBRN (chemical, biological, radiological and nuclear) weapons | **🗴** | "In many disaster deployments, pharmacists have frequently been turned to for expertise in CBRN situations. It is imperative that disaster pharmacists are knowledgeable and know how to access antidotes in all these areas."  "possible drug interactions, administration issues"  "But this is a role for specialist pharmacist, not general pharm"  "Pharmacist should be included in group responsible for these tools - but not sole responsibility for the development of them"  "Specifically, around the use of medicines"  "Whilst pharmacists COULD do this with appropriate training, I think it is a lesser priority and would be considered EXTENDED scope (additional to the recognised scope of practice for the profession) for pharmacists rather than expanded scope (working at top of licence)" |
| Maintain systems and process for the reconciliation and security of controlled drugs (e.g. morphine, oxycodone) | ✓ | "Pharmacists might supply expertise in the subject" |
| Have systems in place to secure cold chain lines | ✓ | "Systems must be in place and absolute limits of each medication in the cache must be documented"  "Not a pharmacist role" |
| Develop a list of at-risk patients in their community | ✓ | "only highly knowledgeable ones"  "Privacy is an issue. Not sure about having a list of individuals prior to an emergency"  "They could add to this list, but I don't think that this would be done by the pharmacist in isolation."  "In collaboration with health providers"  "but must comply with agreed data protection and privacy laws and practices" |
| Response - action in disaster/emergency | | |
| Coordinating logistics of medications and medical supplies for patients with chronic diseases | ✓ | No comments made |
| Rationing limited supplies of medications | ✓ | No comments made |
| Assisting with the release and allocation of national stockpiles if required in pandemic or emergency | ✓ | "Yes, in context that this aspect is one for specialist pharmacists"  "The national commissioning body has a role in this, but pharmacists can play an advisory role" |
| Triage of low-acuity patients. (e.g. medication reconciliation, patient medical history, referring to physician for further assessment or to pharmacist for refill of lost medications) | ✓ | "depending on local policies and practices"  "No sure pharmacists have those skills unless training has been provided" |
| Institute cardiopulmonary resuscitation (CPR) | **🗶** | "Pharmacists should be qualified first aiders and be competent in CPR"  "but pharmacist should be able to perform CPR if no other qualified personal is available"  "Would hope most/all have First aid training and already should be doing this."  "everybody should be able"  "Sad. Everyone should be trained in CPR"  "I think every individual should be trained in this and then there is no difference if one is a pharmacist or not. the education of being a pharmacist doesn't make a difference here"  "Again, this is a role that the pharmacist could do if needed, but probably should sit with another health care professional." |
| Provide wound care and first aid for minor ailments | ✓ | No comments made |
| Providing one off medication emergency supply refills for up to 30 days during the declared disaster | ✓ | "Subject to conditions" |
| Continue provision of chronic disease medications | ✓ | "Subject to conditions" |
| Dispense medications and other necessary medication-related items to affected members of the community (prescription, over-the-counter medications, inhalers) | ✓ | No comments made |
| Dispense general health pharmacy items to affected members of the community (toiletries, nappies, bandages, incontinence pads, water) | ✓ | "anyone can do it"  "particularly payment for this is available for pharmacists via whatever source might exist in local arrangements"  "Unless this is not available elsewhere." |
| Making therapeutic substitutions for drugs available on limited formularies without prior authorisation | ✓ | No comments made |
| Making dose adjustments to existing therapeutic regimens where clinically necessary | **🗶** | This role still had not reached consensus with the use of a 4-point Likert scale. It will be re-queried in the final round with a ‘yes or no’ option for removal and comments |
| Counselling patients on how to use and take medications | ✓ | "Need for some specific CPD [continuing professional development] preparation"  "There is some evidence in literature on pharmacists undertaking such role" |
| Prescribing and administering vaccinations (e.g. tetanus, antidote/prophylaxis to bio-terrorism agent following state public health disaster protocols) | ✓ | "Following protocols"  "a primary role on most disaster deployment"  "But only if there are enough pharmacists employed."  "Need to be careful not to overburden pharmacy professionals with too many roles in disasters and emergencies and to be realistic in what can be done within A health system" |
| Attend clinical ward rounds to provide pharmacist expertise on medical patients | ✓ | "if trained to do so"  "This fits with the role of hospital pharmacists" |
| Prescribe medication needs of low-acuity patients in hospital | ✓ | "clear guidelines and procedures are needed"  "May need this in non-disaster. Introducing NEW procedures can add new problems and not achieve hoped for savings" |
| Medication identification and safety assessment | ✓ | No comments made |
| Monitoring the chronic disease(s) of at-risk individuals to minimise exacerbation | ✓ | "This seems to be part of the ‘list of at-risk patients’ actions"  "not certain here - difficult - depends on local circumstances and policies and practices”  "Pharmacists can be useful in this - especially with point of care diagnostic tools now available"  "Pharmacy professionals are well placed to advise on medications and side effects"  "Pharmacists' role in chronic disease management is standard practice regardless of whether this is focused on minimising exacerbations in the event of a disaster or not." |
| Advocate pharmacy’s role during an event | ✓ | "Including to pharmacists" |
| Maintain media liaison on medication issues | ✓ | "But don't need 100 different experts with differing opinions" |
| Decide on the appropriateness of donated medications and other supplies | ✓ | "Believe this is a WHO recommendation. Needs to comply with WHO and National policies" |
| Pharmacists should engage the pharmacy student workforce to backfill duties (dispensing, inventory), freeing up pharmacists to perform more clinical roles in a disaster. | ✓ | 4-point Likert scale used as was a new role added into the second round. Comments were asked in the final round for this role. |
| Recovery - returning to 'normal' business and beyond | | |
| Provide Mental Health support | **🗶** | "rather than remove it I think we should look to upskill the profession in this area in general and specifically with respect to post-emergency population needs"  "No strong feeling. Feel is a responsibility for staff"  "Many pharmacists have strong relationships with their patients and can certainly provide emotional support as well."  "Further training would be needed to provide this."  "leave it to professionals"  "Not a pharmacist's core training - other specialist better suited to this - but pharmacists working in these settings should be trained and aware of basic mental health support mechanisms" |
| Check on the health needs of the local community | ✓ | "Pharmacists are the third largest healthcare workforce and are well placed to assist public health professionals in identification of population health needs"  "Whatever this means" |
| Re-establish normal stock levels, destroy contaminated stock appropriately | ✓ | "not their role - use techs [technicians]" |
| Restock emergency/ disaster kits for next disaster event | ✓ | "not their role - use techs [technicians]" |
| Identify and prioritise vulnerable patients in local community | ✓ | "particularly if I compliance with ethical and data protection agreements, policies and practices"  "in collaboration with other health providers"  "partially"  "If this feeds into a broader management protocol" |
| Restore order to patient records and drug records, if manually written due to power outages | ✓ | No comments made |
| Document what worked and what did not in the disaster response and change disaster plans accordingly | ✓ | "Absolutely" |
| Participate in post-disaster research/reports | ✓ | No comments made |
| Inform local disaster management reports on pharmacy response improvements | ✓ | No comments made |
