# Supplementary material for: Defining pharmacists' roles in disasters: A Delphi study
Source: PLoS One. 2019 Dec 26;14(12):e0227132. doi: 10.1371/journal.pone.0227132 (PMC6932796; doi:10.1371/journal.pone.0227132)
Supplement: S3 Table — (DOCX) [file pone.0227132.s003.docx]

S3 Table: Final round Delphi study qualitative comments on roles which had reached consensus for pharmacists’ roles across the disaster PPRR phases utilising an all-hazard approach

| **Roles** | **Final comments made** |
| --- | --- |
| **Prevention/Mitigation - reduce the health risks posed by hazards** | |
| Administer vaccinations to prevent diseases in disasters | “It depends on the vaccination. There is no reason that these should not be done by medically trained staff i.e. the pts GP”  “Clearly effective use of resources would be important. If lots of dispensing needed and no other personnel able to do this, but others available to vaccinate, safe use of resources would suggest PhC [primacy health care] dispensing.”  “The comments may reflect confusion over what the researchers expected re: the timing of pharmacists giving such vaccinations. For example, is the question meant to imply pharmacists administering vaccinations prior to any possible future disaster to prevent disease OR giving vaccinations at the immediate time of a disaster to prevent disease outbreak.”  “I agree. This is an important role and one that pharmacists already undertake.”  “I believe this will be an important contribution that appropriately trained pharmacists can offer in these situations.” |
| Educate the public on reducing the spread of communicable diseases/infections | “Ok”  “Role seems clear. Clarifying consistent source of info is critical.” |
| Tailored 'point of care' messaging to chronic disease patients | “OK as well as the GP.”  “None [comments]. Agree.” |
| Ensuring patients are aware of their increased risk of adverse health outcomes in a disaster | “OK as well as the GP.”  “Pharmacist's regular contact with patients when they are well or relatively well should mean a good basis for this type of communication. (This statement, of course assumes that the Pharmacist does actually speak to the patient when they are in the pharmacy, and not completely isolated and only contact is when a script is dispensed.)”  “It would be important to ensure patients are aware of the pharmacist role in this area.” |
| Optimising medication supplies for chronic disease management | “Ok”  “Does seem clear, but some specific training is likely to be needed to assist with anticipating how prolonged shortages are likely to be and consistently prioritising.” |
| **Preparedness - ensure timely and effective response systems are in place** | |
| Ensuring uninterrupted supply of medications in a disaster | “Ok.”  “Many aspects are outside the ability of an individual pharmacist to control. They need adequate information and information pathways to know what is achievable.”  “Obviously this is absolutely a goal at all times, but practical realities of maintaining supply in a disaster may mean this is unachievable... stock availability even in normal times is often difficult! e.g. EpiPen supply problems.”  “This should absolutely be a role of pharmacists - being part of the local and national planning processes will be imperative for pharmacist’s ability to deliver on this.”  “‘Pharmacists should make efforts to avoid interruption to supplies of essential medicines' would be better - some things will be out of our control.” |
| Knowing how to access national stockpiles if necessary | “Essential.”  “They need to know how any stockpile system would work and how it is activated.”  “Yes of course - but in partnership with the emergency plan managers and response networks.”  “Surprised that there is a comment of 'strongly disagree'.” |
| Develop business continuity plans that include disaster management to ensure sustainability of service | “Ok.”  “This seems like a risk mitigation and quality management must. But training and guidance are needed.”  “This is mentioned in most guidance.” |
| Developing drug algorithms and treatment guidelines to determine drug choice based on co-morbidities in the event of bio terrorism (e.g. Anthrax, Plague, Tularaemia - requiring antibiotics/prophylaxis measures) | “This is a medical issue and should be left to clinicians.”  “This seems a specialised role. Clearly one for a, but not necessarily all pharmacists. For the expert pharmacist/s to liaise with other pharmacists would be important.”  “I see this as a specialised role that pharmacists could do but may not be a first priority or a skill-set of all pharmacists.”  “Surprised at the 20% negatives. Pharmacists should clearly part of the team creating treatment algorithms. It is also critical that the pharmacists are experienced in disaster field operations.”  “therapeutic guidance on treatment choices (particularly medication related) in the presence of co-morbidities is a key area that pharmacists can add value, so it is important that they are involved in work such as this.”  “This role is unlikely to be feasible for pharmacists to do in existing resources of training, expertise and funding.”  “There is no reason pharmacists can't apply an algorithm to a clinical problem - dosage calculation is the primary example of this.” |
| Being a part of local/state/national disaster preparedness health meetings - providing medication management advice | “Agree.”  “Agree.” |
| Being a part of the local community disaster management teams to involve pharmacy in coordinated response | “Agree.”  “Yes, agree.” |
| Maintain systems and process for the reconciliation and security of controlled drugs (e.g. morphine, oxycodone) | “Ok.” |
| Have systems in place to secure cold chain lines | “Essential for any facility.”  “They need systems to be able to ATTEMPT to secure cold chain. Some aspects are likely to be outside their control. Possible equally important is a process to be able to differentiate which substances will remain active for how long in case of cold chain failure.”  “technician role.”  “Surprised to see comment 'not a pharmacist role'?? Maintaining the cold chain for vaccine stock is a critical and fundamental role of pharmacists responsible for supply.”  “In addition to this being the right thing to do, and pharmacists are relied upon by others in the healthcare team to be in charge of cold chain (they are relying on pharmacy to be looking after this regardless of whether it is an emergency situation or not - regardless this is a good sensible business decision/action.”  “Agree, yes.”  “Medical logistics are an integral part of the pharmacist's role.” |
| Develop a list of at-risk patients in their community | “In collaboration with GP's and other clinicians.”  “disagree - labour intensive and not key role.”  “Clearly a multidisciplinary role. Privacy concerns people have alluded to need to be considered. Some examples of type of situation/person would help rationalise discussion. May be that a "conditions" list may be more practical than persons list. Does a list of "risk patients" include being aware of who is likely to try to hold up the pharmacy if drugs are in short supply? (i.e. risk to the pharmacy).”  “I agree privacy is an issue - but if done in partnership with others from the disaster risk reduction and management community and most importantly with patient (or family) consent and with proper data confidentiality then this would be very helpful.”  “not pharmacy role – administrative.”  “Can see the intent of this, but I think the comments highlight the practical realities of this. “  “many may have a role in this - but pharmacists are a key part for example in New Zealand those patients who are registered on the community pharmacy LTC [long term conditions] service would be an excellent starting point.”  “Ideally so but there are many challenges to overcome to do this in practice.”  “Part of a team effort.” |
| **Response - action in disaster/emergency** | |
| Coordinating logistics of medications and medical supplies for patients with chronic diseases | “Ok.” |
| Rationing limited supplies of medications | “In collaboration with clinicians only.”  “Need to participate in a coordinated system.”  “political decision.”  “Would likely need to be guidelines in place to ensure equitable and prioritised supply in accordance with need.”  “Critical role in early stages of major disasters.”  “This is an extremely important role, it worries me that some people might disagree with this - if local pharmacists don't assist with this, I'm not sure who could. whilst the national systems work to push medicines into local areas there will be a need for someone to carry out that prioritisation/rationing role locally.”  “Agree.”  “This sometimes happens when short-term medicine shortages occur - a week's supply at a time may be given instead of a month's supply.” |
| Assisting with the release and allocation of national stockpiles if required in pandemic or emergency | “Ok.”  “Potential role for some pharmacists.”  “I think there is a role for the commissioning body, specialised pharmacists and generalist pharmacists.”  “Agree. Specialist pharmacists could advise national commissioning bodies on this.”  “We.ve done it before with Tamiflu and MMR vaccine - totally support.” |
| Triage of low-acuity patients. (e.g. medication reconciliation, patient medical history, referring to physician for further assessment or to pharmacist for refill of lost medications) | “In collaboration with clinicians.”  “I think this is what pharmacists should already be able to do - it worries me that some respondents think pharmacists would need extra training to do this.”  “Training needs to be provided and some form of quality control.” |
| Provide wound care and first aid for minor ailments | “Only if minor.”  “Nursing role.”  “This is a natural extension/continuation of a role that community pharmacists already perform, and this could assist in minor wounds and ailments exacerbating and putting extra pressure on higher level health services.”  “Pharmacists would need training and a mandated role to do so.”  “Strongly agree.” |
| Providing one off medication emergency supply refills for up to 30 days during the declared disaster | “Ok.” |
| Continue provision of chronic disease medications | “Only if as per #24 above [Pharmacists role in providing one off medication emergency supply refills for up to 30 days during the declared disaster].”  “Yes but depends on maintaining updated registers of chronic disease patients.” |
| Dispense medications and other necessary medication-related items to affected members of the community (prescription, over-the-counter medications, inhalers) | “OK if bound by #24[Pharmacists role in providing one off medication emergency supply refills for up to 30 days during the declared disaster].”  “How does this differ from the normal situation? Is this situation making it clear the pharmacist is expected not to abandon ship in a disaster?” |
| Dispense general health pharmacy items to affected members of the community (toiletries, nappies, bandages, incontinence pads, water) | “Ok.”  “The comment regarding source of funding seems particularly relevant.”  “Technician role (or supermarket).”  “Agree that this need not need to be a pharmacist but can be, especially if usual community pharmacy channels are available.” |
| Making therapeutic substitutions for drugs available on limited formularies without prior authorisation | “Doctors don't want this and the argument to free up drs [doctors] is fallacious.”  “Subject to agreement to such a system prior to the emergency.”  “Pharmacists have exactly the skill set required to deliver on this role, and it would assist in freeing up local medical practitioners and nursing staff to spend more time on the wound triage and diagnosing roles/emergency hands on roles that they are more expert in.”  “Governance procedures are needed for quality control and monitoring right substitutions for drugs were made.”  “Strongly agree.” |
| Counselling patients on how to use and take medications | “Ok.”  “I don't agree with previous comments made that pharmacists might need specific CPD preparation - counselling patients on how to use and take medications is core business for pharmacists. I agree that pharmacists should do CPD preparation about what's different in an emergency/how to manage in an emergency - but is different to this question I think.”  “Training is needed to do so.” |
| Prescribing and administering vaccinations (e.g. tetanus, antidote/prophylaxis to bio-terrorism agent following state public health disaster protocols) | “Only in collaboration with doctors and only for e.g. ADT [diphtheria and tetanus vaccine].”  “Realistic use of available limited resources is important.”  “Pharmacists trained to give vaccination now should be able to provide vaccination services in a disaster situation.”  “This is another team effort. At the least, the disaster pharmacy should oversee the viability, dosage, etc.”  “Agree.”  “After training has occurred.” |
| Attend clinical ward rounds to provide pharmacist expertise on medical patients | “Ok.”  “No, they will be busy enough on priority matters.”  “Training is needed. Hospital pharmacists can do so.”  “If trained.” |
| Prescribe medication needs of low-acuity patients in hospital | “No - only if as per #24 [Pharmacists role in providing one off medication emergency supply refills for up to 30 days during the declared disaster] and only in collaboration with clinicians.”  “Particularly useful in the situation of an exacerbation of an on-going condition or primary care level acuity - guidelines would be useful.”  “This should become a role in non-disaster situations to ensure the procedures and infrastructure needed are there for it to happen in disaster situations.” |
| Medication identification and safety assessment | “Ok.” |
| Monitoring the chronic disease(s) of at-risk individuals to minimise exacerbation | “In collaboration with pts [patients] doctor(s).”  “where resources are available and as part of the main emergency plan of the health care and emergency responders as agreed in advance.”  “difficult to do.”  “Agree with comment: "Pharmacists' role in chronic disease management is standard practice regardless of whether this is focused on minimising exacerbations in the event of a disaster or not.".”  “The role that pharmacists already play in this area can have a significant positive impact on the resilience of local communities and help people to self-manage and thereby reduce the pressure on more specialised health services.”  “Pharmacists can offer advice if requested from patients but a difficult one in practice due to confidentiality and data sharing pharmacists may not be able to keep registers of high-risk patients.”  “As part of a team.” |
| Advocate pharmacy’s role during an event | “Ok.”  “Glad this one got consensus!”  “Completely agree.” |
| Maintain media liaison on medication issues | “Ok.”  “Their media liaison probably needs to be limited to areas where they will be recognised as experts - i.e. in relation to medicines (etc).”  “where requested and in partnership with the emergency plan managers and implementers.”  “Where directly applicable but in times of disaster there should be central points of communication to ensure consistency and accuracy of message.”  “they certainly need to be in the loop and I agree with the comment that we don't need 100 different experts - it would need to be well managed, agreed spokes people and good communication out to all practitioners so that the messaging is consistent (local pharmacists need to be part of those national planning meetings so they know how to make sure they are kept in the loop).”  “Local PGA/PSA [pharmacy guild of Australia/pharmaceutical society of Australia] reps [representatives] have probably been trained and would be the ideal focus.” |
| Decide on the appropriateness of donated medications and other supplies | “In collaboration with clinicians.”  “Agree need guidelines to ensure appropriateness.”  “There are specific national bodies which should do so.” |
| Pharmacists should engage the pharmacy student workforce to backfill duties (dispensing, inventory), freeing up pharmacists to perform more clinical roles in a disaster. | “Ok.”  “Providing standards can be maintained. It doesn't matter how good the pharmacists’ clinical input is if the prescribed medicine doesn't get to the patient.”  “Agree.”  “As stated in my previous response... this is dependent on the year level and student competence. I don't think one can simply say, 'all students'.”  “There are plenty of roles that pharmacy students, technicians, pharmacy assistants could fulfil - we should have a good honest look at what really needs the pharmacist expertise. Those members of these groups who already have work experience of how the supply chain works/records required could add huge value around supporting processes and procedures.”  “Complicated in practice.”  “Strongly agree.” |
| **Recovery - returning to 'normal' business and beyond** | |
| Check on the health needs of the local community | “In collaboration with clinicians.”  “The role would need to be clarified prior to a disaster.” |
| Re-establish normal stock levels, destroy contaminated stock appropriately | “Responsibility for role should sit with the pharmacist. Tech may be useful on logistic process. For techs to undertake some roles would require change to legislation.”  “tech [technician] role.”  “Agree that this need not necessarily be a role of pharmacists, and the technician workforce could do this.”  “I agree pharmacists have a role to play but so too do techs and other pharmacy staff.” |
| Restock emergency/ disaster kits for next disaster event | “Responsibility should sit with pharmacist. If the role can safely be delegated to appropriately trained techs, that is fine.”  ““tech [technician] role.”  “Agree that this need not necessarily be a role of pharmacists, and the technician workforce could do this.” |
| Identify and prioritise vulnerable patients in local community | “In collaboration with other health care providers.”  “Yes but need to comply with data protection guidance.” |
| Restore order to patient records and drug records, if manually written due to power outages | “Only for dispensed medications.” |
| Document what worked and what did not in the disaster response and change disaster plans accordingly | No additional comments |
| Participate in post-disaster research/reports | No additional comments |
| Inform local disaster management reports on pharmacy response improvements | No additional comments |
