# Supplementary material for: Defining pharmacists' roles in disasters: A Delphi study
Source: PLoS One. 2019 Dec 26;14(12):e0227132. doi: 10.1371/journal.pone.0227132 (PMC6932796; doi:10.1371/journal.pone.0227132)
Supplement: S4 Table — (DOCX) [file pone.0227132.s004.docx]

S4 Table: Final round Delphi study survey on roles which had not yet reached consensus in the previous two rounds and included the comments from the panellists

| **Role** | **Consensus reached** | **Keep /Remove** | **Comments for and against role** |
| --- | --- | --- | --- |
| Develop educational tools for health professionals on preparedness, signs and symptoms and drug treatments for CBRN (chemical, biological, radiological and nuclear) weapons | **🗶** | Keep = 9 (60%) | “They are medicines - we need to know about their use and provision.”  “It is an important role for SOME (not all) pharmacists. May fit well with pharmacists working for example in Poisons Information Centres, and clearly important for those in Defence Department positions.”  “In partnership with others in preparing emergency plans and in collaboration with the overall training programme where linked.”  “I reiterate what I think was my own comment in last round: "Whilst pharmacists COULD do this with appropriate training, I think it is a lesser priority and would be considered EXTENDED scope (additional to the recognised scope of practice for the profession) for pharmacists rather than expanded scope (working at top of licence)".”  “agree with the comments about having more specialised pharmacists involved in this, but also for them ensuring that the knowledge is disseminated out to other pharmacists.”  “But in collaboration with other health professionals.” |
|  |  | Remove = 6 (40%) | “Community Pharmacists would require considerable training to undertake this role.”  “not pharmacist role.”  “Pharmacists could be included in specialist committees which develop such guidance (for example, NICE).” |
| Making dose adjustments to existing therapeutic regimens where clinically necessary | **🗶** | Keep = 10 (66.67%) | “This is a POTENTIAL role, but training and agreement from other professions needed.”  “If agreed in principle in advance depending on training and expertise.”  “Cognitive role.”  “This is a broad statement and guidance on what circumstances this might apply needs to be provided. e.g. dose adjustment of aminoglycosides/anticoagulants in response to TDM [therapeutic drug monitoring].”  “Part of requirement to be an effective disaster pharmacist.”  “Would need therapeutic guidance to be disseminated - this would be a good thing to disseminate in any event i.e. to support routine practice.”  “In conjunction with work protocols.”  “But within clear protocols.”  “Protocols and calculations - primary pharmacist skills.” |
|  |  | Remove = 5 (33.33%) | “Community Pharmacist require further training to fully interpret Lab test results and the impact on dose adjustments.”  “This is a doctor's role.” |
| Institute cardiopulmonary resuscitation (CPR) | **✓** | Keep = 13 (86.67%) | “All Australians should know how to do CPR.”  “I thought there was earlier agreement to First Aid provision. I could be wrong, but last time I did a first aid course it included CPR. NT [Northern Territory] legislation REQUIRES any trained first aider to provide first aid.”  “Pharmacist should be able to perform CPR if no other qualified personal is available."  "Pharmacists should be qualified first aiders and be competent in CPR"  “No doubt at all!”  “If a pharmacist is working in a public facing role especially where they are the highest qualified present (i.e. community pharmacy) they should be able to do this. There is some clinical institution based advisory roles where those pharmacists are specifically excluded from hands on roles - I would view these differently if others are present. But overall bottom line is that regardless of whether someone is a health professional or otherwise we would expect assistance, wouldn't we?”  “As previously said, everybody should have a role in CPR, however more likely to be performed by the crash team.”  “Training is essential.”  “Only if no other health Professionals available.”  “Training needed.” |
|  |  | Remove = 2 (13.33%) | No comments made |
| Pharmacists role in providing behavioural and mental health support following a disaster to their patients, customers and staff | **🗶** | Keep = 8 (53.33%) | “feeding back to the skilled workforce.”  “I feel this is currently outside scope of existing training, but aware increasing number of pharmacists taking interest. I would prefer to abstain from this answer. If I have to respond, is qualified yes depending on training, other roles needed etc.”  “if agreed and trained in advance and documents.”  “Frequently, patients with behavioural challenges have been separated from their medications. Pharmacist is important in supporting stabilizing”  “Further training is needed.” |
|  |  | Remove = 7 (46.67%) | “Pharmacists have insufficient clinical knowledge and training to undertake such roles.” |
